# Sequential Bilateral Peripheral Nerve Stimulation of Lumbar Medial Branches Following Laminectomy: A Case Report

**DOI:** 10.7759/cureus.71850

**Published:** 2024-10-19

**Authors:** Daniel Briggi, Colton Reeh, Gaibo Yan, Christian Vangeison, Emanuel N Husu

**Affiliations:** 1 Physical Medicine and Rehabilitation, Baylor College of Medicine, Houston, USA; 2 Family Medicine, The University of Tennessee Health Science Center, Knoxville, USA; 3 Pain Medicine, The University of Texas Health Science Center, Houston, USA; 4 Pain Medicine, Baylor College of Medicine, Houston, USA; 5 Clinical Sciences, Rosalind Franklin University of Medicine and Science, Chicago Medical School, North Chicago, USA

**Keywords:** 60-day pns, axial low back pain, failed back surgery syndrome (fbss), peripheral nerve stimulator, temporary pns

## Abstract

In a patient with a history of lumbar spine surgery, the management of refractory low back pain may prove challenging. Advancing neuromodulatory techniques and technologies offer the promise of significant and sustained relief in these patients, though prior literature is limited. We present a case of peripheral nerve stimulation at the lumbar medial branches in a woman with chronic axial low back pain following a three-level laminectomy. Six months after the sequential bilateral treatment, the patient reported complete relief of her low back pain, increased activity, and a significant reduction in oral medication burden.

## Introduction

Approximately 500,000 lumbar spinal surgeries are performed in the United States annually, and the number of cases is steadily increasing each year. An estimated 10% to 40% of surgery cases result in failed back surgery syndrome (FBSS), a debilitating complication characterized by persistent lumbar spine pain despite surgical intervention for pain in the same topographical location [1]. The etiology of FBSS is complex and likely multifactorial, with structural, neuropathic, psychosocial, and surgical causes [2]. Conservative treatment is the recommended initial approach, including physical therapy and oral medications such as acetaminophen, non-steroidal anti-inflammatory drugs, gabapentinoids, antidepressants, muscle relaxants, and opioids. For pain refractory to conservative treatments, more aggressive management including injections, surgery, and implantable technologies may be considered. A literature review demonstrates weak evidence for the long-term efficacy of medication-based and surgical approaches to FBSS, with stronger evidence for implantable technologies [3,4].

Current implantable technologies include spinal cord stimulation (SCS) and intrathecal drug delivery systems (IDDSs). However, SCS requires leads to be placed in the epidural space and may be contraindicated in patients with pacemakers, coagulopathy, and infection risks [5], and IDDSs require catheter placement in the thecal sac and may have similar contraindications. A less invasive and alternative implantable option is peripheral nerve stimulation (PNS), which may be temporary or permanent. This minimally invasive, drug-free intervention involves placing an electrode near a peripheral nerve of interest and stimulating that nerve to reduce pain and improve function. Recent studies have shown temporary PNS to be an effective treatment option for chronic low back pain and disability [6,7].

While the mechanism of pain relief with PNS is not entirely clear, the explanation may be multifactorial. As with other neuromodulatory systems, PNS could alter synaptic transmission through gate control [8]. By reducing symptoms for a period of weeks to months, this treatment may permit increased activity and central remodeling, reducing the impact of sensitization as a contributor to experienced pain. In the case of lumbar medial branch stimulation, though, the activation of efferent motor fibers to local multifidi may also offer proprioceptive [9] and stabilization benefits [10].

With informed consent, the authors present the case of a woman with a complex medical history including FBSS who underwent a sequential implantation of temporary 60-day PNS devices targeting the bilateral L4 medial branches at the L5 vertebral bodies, yielding 100% low back pain relief and a dramatic reduction in medication burden.

## Case presentation

A 63-year-old female with a past medical history notable for anxiety and depression, alcohol use disorder, osteoporosis, and lumbar spinal stenosis status post-L3-L5 laminectomy at an outside hospital four years earlier presented for the evaluation and management of midline, constant, low back pain rated 8/10 on the Visual Analog Scale (VAS, from 0, no pain, to 10, worst pain) with radiation to her posterolateral left leg. Though she had initially experienced relief from the prior lumbar decompression, her pain had since returned. Symptoms failed to improve with prior physical and aquatic therapies. On presentation, she was taking duloxetine 30 mg daily, gabapentin 600 mg three times daily, cyclobenzaprine 10 mg every six hours as needed, and acetaminophen with codeine 300-30 mg every four hours as needed. Initial physical examination was notable for 5/5 strength and grossly intact light touch sensation in the bilateral lower extremities, symmetric 2+ patellar and Achilles reflexes, and reproducible pain with palpation of the left lumbosacral paraspinal muscles, left superior gluteal nerve, and bilateral posterior superior iliac spines. She had a positive Kemp’s test, a positive left sitting flexion abduction external rotation test, and a negative bilateral slump test.

**Figure 1:**
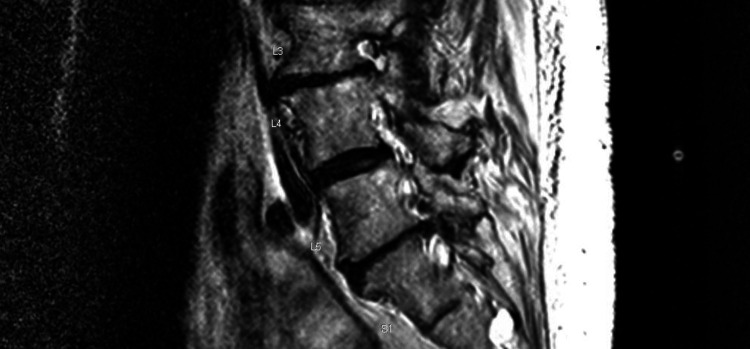
Sagittal MRI with facet arthropathy and L4-L5 anterolisthesis.

**Figure 2:**
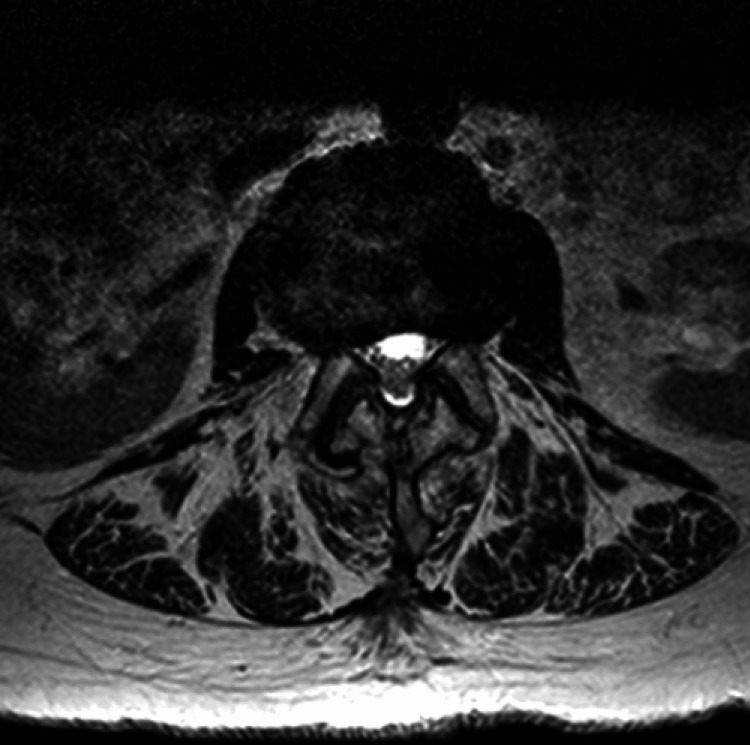
Axial MRI with L4-L5 facet joint space narrowing and osteophyte formation.

The patient underwent a left sacroiliac joint corticosteroid injection under fluoroscopy one week after the initial evaluation with fair local symptom relief but persistent low back and right flank pain. Magnetic resonance imaging (MRI) of her lumbar spine with and without contrast revealed advanced multilevel disc degeneration, severe right and moderate left foraminal stenosis at L2-L3, and severe facet arthropathy at the L4/L5 level with grade 1 anterolisthesis of L4 on L5 (Figures 1, 2). Subsequently, she underwent a right lumbar medial branch block that yielded 70% pain relief.

**Figure 3:**
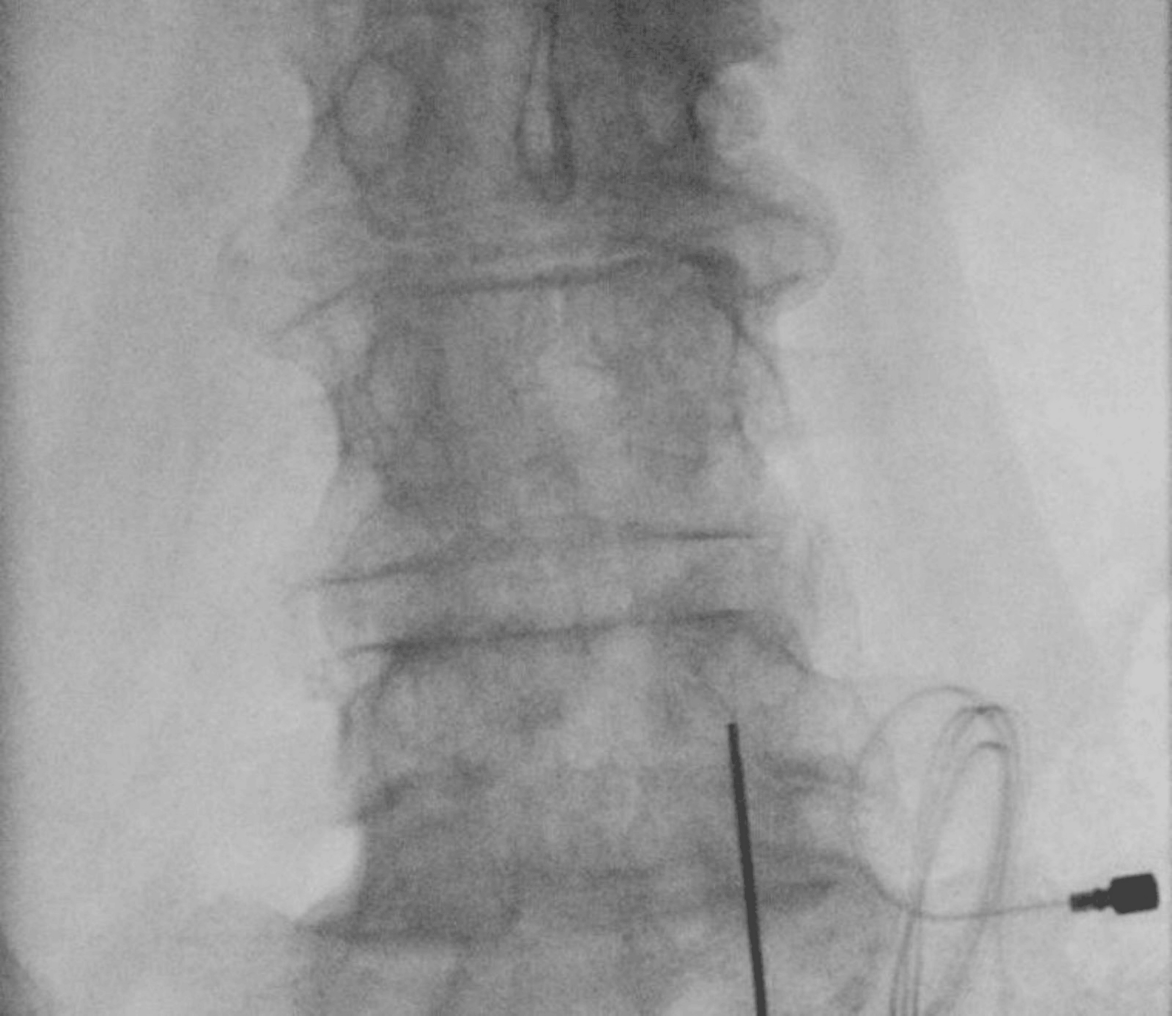
Peripheral nerve stimulator placement at the right L4 medial branch.

**Figure 4:**
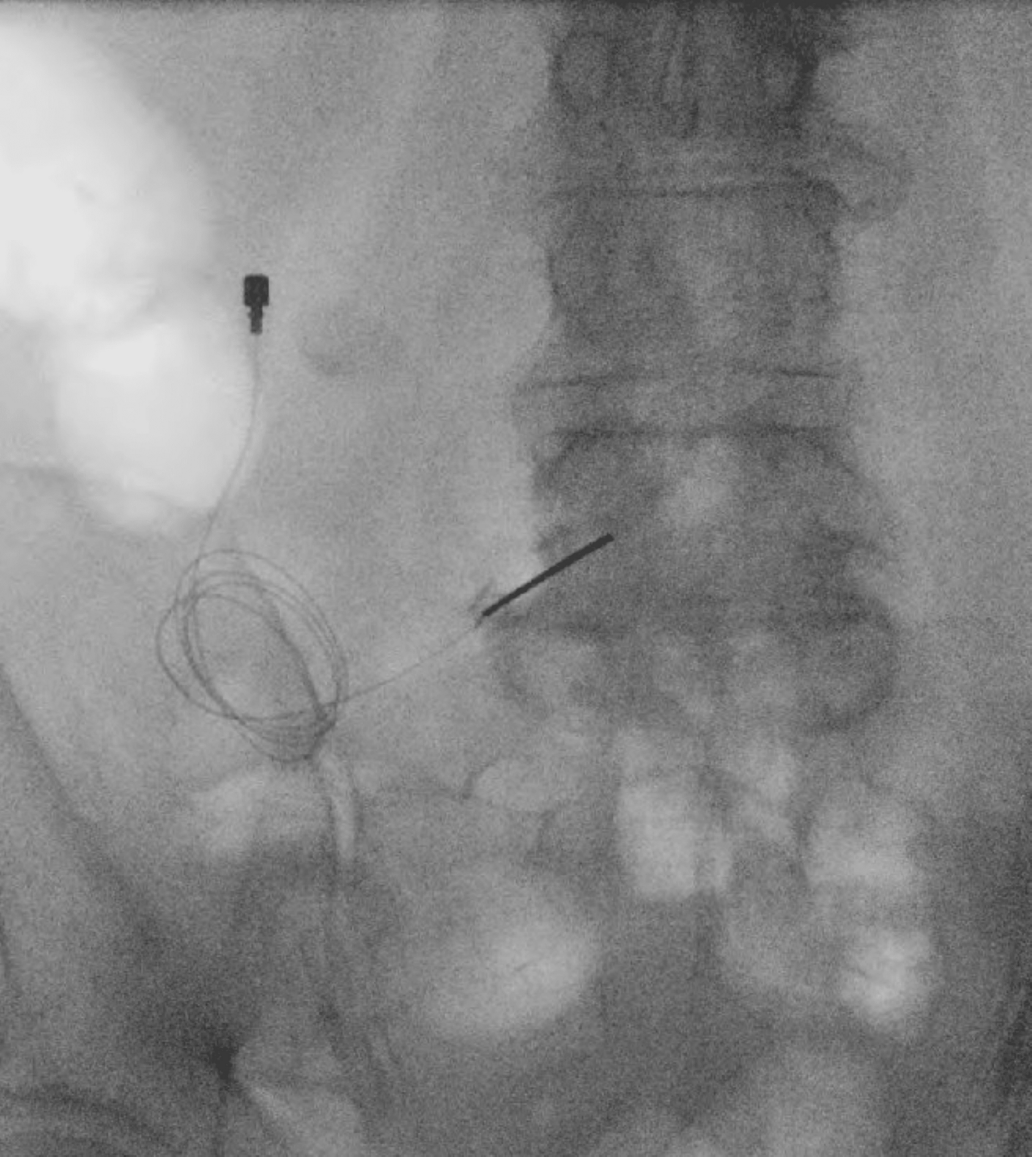
Peripheral nerve stimulator placement at the left L4 medial branch.

Twelve months after her initial evaluation and constrained in her treatment options by the payor, the patient underwent careful implantation of her first temporary PNS lead for persistent low back pain attributed to facet arthropathy. The right L4 medial branch was targeted at the L5 vertebral body under fluoroscopic and ultrasound guidance. Revision (Figure 3) was required one month later due to an unintentional explant by the patient, and this revised lead was removed at 60 days with concurrent placement of left L4 medial branch PNS at the L5 vertebral body (Figure 4). At the time of the left-sided implant, the patient had experienced 80% relief of her central low back pain.The left-sided implant was then removed 60 days after its placement without incident. In each case, the external pulse generator was secured at the ipsilateral flank with adhesive.

Six months after the removal of her left peripheral nerve stimulator, the patient reported “complete” relief of her low back and referred left leg pain with a VAS score of 0/10 and resumption of prior daily activities. The patient had discontinued duloxetine, gabapentin, and cyclobenzaprine, and remained off opioids. Her remaining relevant medication regimen consisted of baclofen 10 mg three times daily as needed for muscle spasms.

## Discussion

Though a recent retrospective review [11] has shown temporary PNS to provide meaningful pain relief across a number of nerve targets, literature on temporary PNS as a treatment for low back pain is sparse. In 2020, Gilmore et al. performed temporary PNS stimulation on nine subjects’ lumbar medial branches for a one‐month therapy period, after which the leads were removed. Pain and disability were assessed up to 12 months after lead removal. Most subjects who completed follow‐up experienced sustained, clinically significant reductions in pain (average 63% reduction at one year) and disability (32‐point reduction in disability among responders as measured by the Oswestry Disability Index), as well as improved quality of life [6]. In 2022, Fiala et al. performed lumbar PNS on three male and three female patients (five of the six patients at the L4 level) as a treatment for chronic low back pain, with an average of 46 days of treatment and a 64.8% pain reduction following explant [7]. These studies set the stage for the authors’ selection of treatment modality for this patient.

Throughout the course of this patient’s treatment, she had consistently expressed a preference to pursue minimally invasive treatment options and to avoid neurosurgical consultation given the failure to sustain relief with prior laminectomy. With MRI evidence of adjacent segment disease and good relief from prior medial branch nerve block, the bilateral lumbar facet joints were determined to be this patient’s most significant pain generators, and lumbar medial branch radiofrequency ablation (RFA) had initially been proposed as a treatment option. RFA was denied by her insurance company due to the realization of only 70% relief with diagnostic medial branch block, and given her desire for minimally invasive treatment, the benefits of a temporary and targeted implant were preferred over those of** **a spinal cord stimulator or permanent peripheral nerve stimulator. Additionally, avoidance of lumbar medial branch rhizotomy allowed for the maintenance of motor innervation to the lumbar paraspinals, which may be important in patients who have previously failed to achieve sustained relief through back surgery. While initially intended to be bilateral and concurrent, her treatment was bilateral and sequential due to payor requirements.

PNS does come with the risks of procedure-site pain, infection, lead migration, and lead fracture [12], and contraindications include allergy, coagulopathy, and active infection. Pain and infectious complications, however, are theoretically reduced by the temporary nature of the implant and the external fixation of the pulse generator. Lead migration and lead fracture had been previously reported to be 2% and 1%, respectively [13], and recent technological advancements might be expected to reduce those risks further.

## Conclusions

The patient described in this study experienced complete relief of her low back pain with sequential unilateral implantation of 60-day temporary PNS at the bilateral lumbar medial branches following three-level lumbar laminectomy. She reported subjective pain reduction, increased activity levels, and near-total reduction of oral pharmacotherapy. Future studies may seek to evaluate the impact of PNS as an opioid-sparing therapy and explore the effects of sequential versus concurrent temporary PNS implantation in patients with facet-mediated low back pain.
